# Prospective and Descriptive Study on Serum Androstenedione Concentration in Healthy Children from Birth until 18 Years of Age and Its Associated Factors

**DOI:** 10.1155/2017/9238304

**Published:** 2017-05-16

**Authors:** María Gabriela Ballerini, Virginia Gaido, María Eugenia Rodríguez, Ana Chiesa, María Gabriela Ropelato

**Affiliations:** Centro de Investigaciones Endocrinológicas “Dr. César Bergadá” (CEDIE)-CONICET–FEI–División de Endocrinología, Hospital de Niños Dr. Ricardo Gutiérrez, Gallo 1330, C1425EFD Buenos Aires, Argentina

## Abstract

**Introduction:**

Androstenedione (A4) is an adrenal and gonadal steroid biomarker, useful in the assessment of children in whom steroidogenic disorders are suspected. The first key step in the evaluation of a diagnostic test resides on confident reference intervals (RI). The lack of updated A4-RI with current methods in pediatrics may mislead A4 results and limit its diagnosis accuracy.

**Aim:**

To provide A4 reference ranges in healthy children.

**Methods:**

Prospective, descriptive study. 283 children aged 4 days to 18 years were included. In children < 1 yr, A4 was measured directly in serum (NE-A4) and postorganic solvent extraction (E-A4) for the assessment of interfering steroids. The influence of chronological age (CA), gender, and Tanner stage (T) were investigated.

**Results:**

In the neonatal period, E-A4 was significantly lower than NE-A4; boys had higher NE-A4; sexual dimorphism disappeared after extraction procedure. In children older than 4 months, A4 concentration remained low until the age of 5 years. Thereafter, A4 increased significantly in association with CA and T (*r*^2^ = 0.65; *p* < 0.001), obtaining the highest concentrations in children within pubertal ages without sexual dimorphism.

**Conclusion:**

We recommend to perform solvent extraction in neonates and to take into account age and sexual development to properly interpret A4 results in childhood.

## 1. Introduction

Serum 4-androsten-3,17-dione (androstenedione) provides a useful marker of androgen biosynthesis. In pediatrics, androstenedione (A4) measurement complements the evaluation of steroidogenesis disorders, especially in the diagnosis of hyperandrogenemic syndromes such as premature pubarchy, classical, and nonclassical congenital adrenal hyperplasia (CAH) as well as to monitor these patients during treatment [1]. Since clinical phenotypes of patients with abnormal steroidogenesis may present a wide variability, multiple steroid hormone analyses are usually necessary for diagnosis that could also orientate further molecular studies. Nevertheless, methodological factors may influence its adequate measurement. Immunoassays are susceptible to interferences due to the crossreactivity of other steroids [2]. Although in neonates, the presence of steroids that interfere with serum 17 hydroxy-progesterone (17OHP) measurements has been previously reported using current RIA methods [3–6]; no study has stated this kind of interference for A4 measurement.

Gold standard methods for quantifying A4 and other steroids such as LC-MS/MS technique are not always available in routine laboratories, and for current methods of RIA, little information on reference intervals (RI) in children and adolescents are available. Although the first key step in the evaluation of a diagnostic test resides on confident RI, manufactures do not always provide A4 reference data according to age, gender, and pubertal development from birth to adolescence. The lack of confident RI for A4 in pediatrics in addition to methodological factors may lead to an inappropriate interpretation of A4 results in this period of life, thus lowering its diagnostic usefulness.

This study aimed to evaluate the methodological- and physiological-related variations of serum A4 concentration using a current commercially available RIA method in order to establish RI from birth to adolescence.

## 2. Methods

Serum A4 concentration was measured by a current competitive Androstenedione-RIA-Coated tube kit from DIAsource (DIAsource, Nivelles, Belgium). The study was conducted at the Endocrinology Laboratory of the Children's Hospital Ricardo Gutiérrez of Buenos Aires, Argentina. The protocol was followed exactly as recommended by manufactures. The analytical performance of the A4-RIA method was assessed by using international protocols for method evaluation from the Clinical and Laboratory Standards Institute (http://www.clsi.org/). Commercial control materials from Bio-Rad (Lyphochek® Immunoassay Plus) were used to ascertain intra- and between-run precision (CVi% and CVb%, resp.) and accuracy (bias%); Bio-Rad control lot number 40270 had a mean reported and range for Level 1: 3.39 nmol/L (2.5–4.3 nmol/L) and for Level 2: 12.8 nmol/L (8.9–16.8 nmol/L)]. CVi% and CVb% for level 1 were 4.5% and 9%, respectively; for level 2, 3.7% and 5.9%, respectively. Mean A4 concentration obtained for these controls were 3.25 and 13.3 nmol/L for control levels 1 and 2, achieving a bias for all levels of 4.1% and 3.7% as compared to that of manufactures that were within the reported range by Bio-Rad. Total error (TE) was calculated as follows: TE = (1.96 × %CV) + %bias. The obtained TE was compared to the allowable TE (aTE) based on biological variability (aET = 35.3%). RIA-DIAsource method presents an analytical range of 0.35–30.9 nmol/L. No data on calibration traceability was provided by manufactures. A4 method crossreacts principally to testosterone (0.24%).

To test the presence of interference steroids in children ≤ 1 year of age [3], A4 concentration was measured directly in serum as mentioned above (nonextracted: NE-A4) and after an organic solvent extraction procedure (extracted serum: E-A4) using diethyl ether as we previously described [5]. The efficiency of the extraction procedure was evaluated [5]. Only runs with recoveries above 80% were accepted (87 ± 22% for an androstenedione concentration of 3.5 nmol/L).

## 3. Subjects

Two hundred and eighty-three full-term (FT) healthy neonates, healthy children, and adolescents aged 4 days to 18 years old were included prospectively, taking into account sample size calculated (http://www.apps.who.int/iris/handle/10665/37589), with a confidence level of 95%, an absolute accuracy of ±7%, and assuming a biological variation in population of 35.3% (https://www.westgard.com/biodatabase3.htm).

Samples from children ≤ 1 year of age were obtained from babies who were recalled for abnormal result of neonatal 17OHP or TSH performed at maternity discharge (median 8 days of life) and after a careful clinical and biochemical evaluation was found to be healthy. Children ≤ 1 year were studied according to the following age categories: ≤2 months (Group I (GI, *n* = 48; boys = 33)), 2–4 months (Group II (GII, *n* = 23; boys = 14)), and 4 months to 1 year (Group III (GIII, *n* = 20; boys = 12)). Serum samples corresponding to preterm babies were not included in the present study. Healthy children > 1 year of age included in this work belonged either to the control group of a clinical study on idiopathic short stature in which IGFALS gene variants were evaluated [7] or to children that consulted the Endocrinology Division for presumed thyroid abnormalities during April 2014 to September 2015 and were found to be clinically and biochemically normal. All children underwent a full clinical pubertal assessment done by trained pediatric endocrinologists of the Endocrinology Division of the Children's Hospital of Buenos Aires. Pubic hair in addition to breast development in girls and testicular volume and genitalia features in boys was evaluated in each child to further categorize into the Tanner stage [8, 9]. The children were grouped into prepubertal and pubertal groups (T I and T II-V, resp.). Prepuberty was subcategorized by age according to previous studies into the following age ranges [5, 6, 10]: T Ia (1–4 years); T Ib (girls ≥ 5 to 8 years; boys ≥ 5 to 9 years); and T Ic (≥8 years for girls and ≥9 years for boys). Samples in postmenarchal girls were taken in the early follicular phase (days 3 to 5). The children were not under steroid therapy or other treatments at the time of the study. Sample collection was made within 8-9 a.m. Samples were stored at −20°C until assayed, avoiding freezing and thawing as recommended by manufactures. Samples with hemolysis were not processed.

This study was approved by the local Institutional Review Board of Dr. Ricardo Gutiérrez Children's Hospital, Buenos Aires, Argentina.

## 4. Statistical Analysis

Data distribution of serum A4 concentration was tested for normality using the Shapiro-Wilk test. As A4 did not follow a Gaussian distribution, serum concentration was log-transformed to reach normal distribution. Intersubject variability of circulating A4 concentration in normal children from birth up to 18 years was calculated as (SD/mean)∗100, being SD the standard deviation and the mean of the data of normal children [11]. Spearman correlation, multiple regression analysis and one-way ANOVA followed by Tukey as posttest was used to assess differences in serum A4 concentration by chronological age (CA), gender, and pubertal stage.

Data were analyzed using GraphPad Prism Version 4.00 for Windows (GraphPad Software San Diego, CA; http://www.graphpad.com). Statistical significance was accepted for *p* < 0.05.

## 5. Results

**Figure 1:**
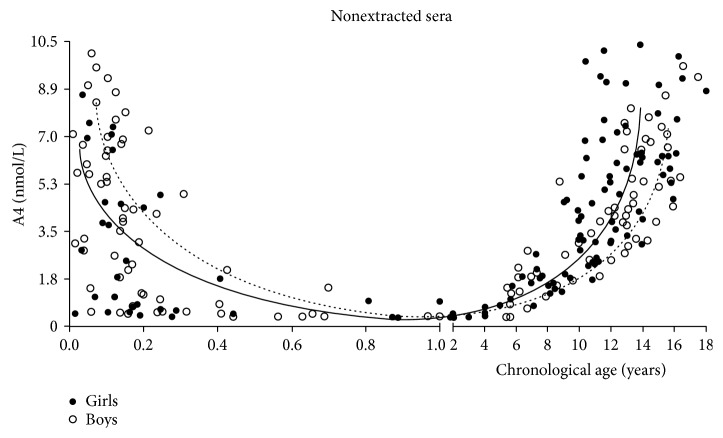
Nonextracted androstenedione concentration in children from birth to adolescence. The curves denote the median NE-A4 concentration for girls (solid line) and for boys (dashed line) throughout childhood.

**Figure 2:**
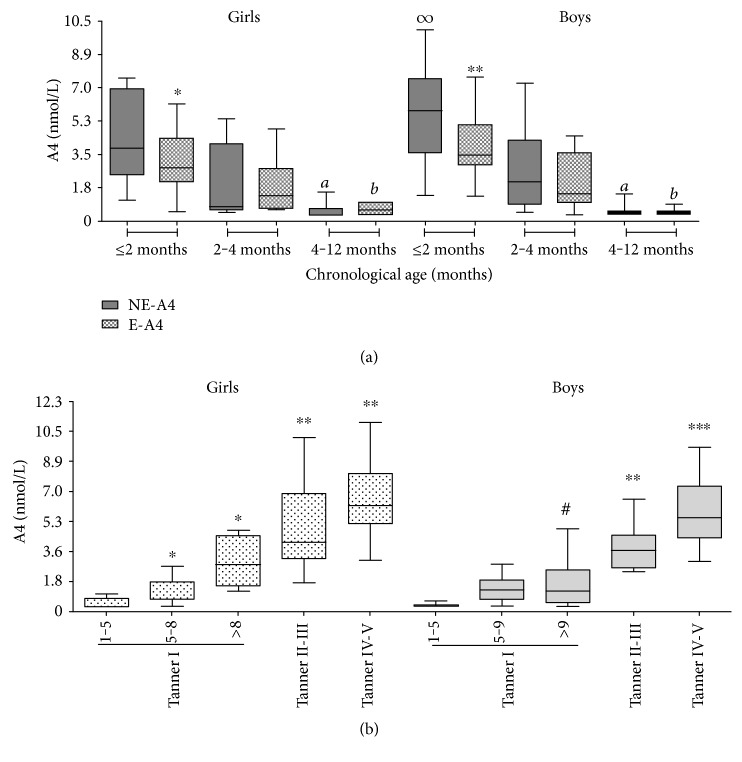
(a) Androstenedione concentration in nonextracted sera (NE-A4, grey box) and after organic solvent extraction (E-A4, dotted box) in girls and boys within the first year of postnatal life. The line denotes the median and the whiskers denote the 2.5 and 97.5 percentiles. ^∗^*p* < 0.05 and ^∗∗^*p* < 0.001 versus NE-A4 concentration in age-matched girls and boys, respectively; ^∞^*p* < 0.05 versus NE-A4 for girls ≤ 2 months; ^*a*^*p* < 0.01 versus NE-A4 in GI within each sex group; ^*b*^*p* < 0.001 versus E-A4 in GI within each sex group. (b) Serum androstenedione concentration in children ≥ 1 year. The line denotes the median and the whiskers denote the 2.5 and 97.5 percentiles. ^∗^*p* < 0.05 versus 1–5 years old girls; ^∗∗^*p* < 0.01 and ^∗∗∗^*p* < 0.001 versus Tanner I within each sex group; ^#^*p* < 0.01 versus girls in Tanner *I* > 8 years.

Nonextracted A4 varied widely throughout childhood achieving an interindividual CV% of 76% (Figure 1). During the first year of life, NE-A4 (*r* = −0.61; *p* < 0.0001) and E-A4 (*r* = −0.58; *p* < 0.001) concentrations decreased in association with CA. Within the first 2 months of life, none of the newborns presented NE- or E-A4 concentration below the limit of quantification of the assay (0.35 nmol/L). Serum A4 concentrations were always higher in NE-A4 samples during the first year of life compared to those in E-A4 samples, reaching the statistical significance in the group of children ≤ 2 months (Figure 2(a)). Boys ≤ 2 months had higher NE-A4 concentration than age-matched girls (*p* < 0.05). Sexual dimorphism observed for NE-A4 samples in GI disappeared after the extraction procedure (*p* = 0.08). Nonextracted A4 and E-A4 were significantly lower in GIII compared to that in GI (*p* < 0.01 and *p* < 0.001, resp.). In children older than 4 months, A4 concentration remained low until the age of 5 (Figure 2(b)). Thereafter, A4 increased in association with CA and T (*r*^2^ = 0.65, *p* < 0.001). A4 concentration changes significantly throughout prepuberty, obtaining the greatest increase at late infancy for both sexes and the highest concentrations in children within peripubertal ages, where a sexual dimorphism with higher A4 concentration in girls were observed. A4 concentration continued rising along pubertal development. Pubertal girls as a whole group presented slightly higher A4 levels than pubertal boys; however, this difference did not reach the statistical significance (median and 3rd–97th centile range: T II–V: 5.9 (2.1–10.2) nmol/L versus boys T II–V: 4.7 (2.3–6.5), *p* = 0.13).

Serum reference ranges in children from birth up to adolescence are presented in Table 1.

## 6. Discussion

Androstenedione is a steroid of adrenal and gonadal origin that completes the assessment of the androgen profile in children in whom steroidogenic disorders are suspected. The present study demonstrates that chronological age and pubertal development strongly influence serum A4 concentration. In addition, some methodological factors related to the presence of interfering steroids in serum should also be considered to improve its diagnostic accuracy during the neonatal period. While LC-MS/MS technique constitutes a more sensitive, specific, and accurate methodology for A4 and for other steroid quantifications [2, 12, 13], this expensive technology is not readily available in routine laboratories, especially in public hospitals. Instead, RIA methods are commonly used in daily practice. However, there are not updated reference data for pediatrics for most current A4-RIA methods, which implies a lower diagnostic efficiency when interpreting A4 results in children and adolescents. Besides the lack of confident reference data for current RIA, the analytical performance of immunoassays may also influence the accuracy of the results. In this sense, and after performing an analytical validation of RIA-DIAsource method using protocols from the Clinical and Laboratory Standards Institute (http://www.clsi.org/), we evaluated the physiological changes of serum A4 concentration with CA, gender, and sexual development in healthy children and adolescents from birth until 18 years of age. Androstenedione concentration varied largely among healthy children. Moreover, it doubled the online data of interindividual variation in adult life (35.3%). To our knowledge, no other study has stated A4 interindividual variation during childhood.

Given our own previous experience on steroid measurements in neonates [5, 6, 14], we investigated the presence of interfering steroids by performing an organic extraction step prior to A4 quantification in children < 1 year. Androstenedione (either NE- or E-A4) concentration was always higher in children within the first 2 months of life for both sexes probably due to the active steroidogenic activity of the gonads [14–16] and the fetal adrenal zone that persists until the first year of postnatal life [10, 17]. Controversial results on A4 concentration and sexual dimorphism were reported in neonates by using different assays [2, 13, 16, 18, 19]. In accordance to Forest and Cathiard [16] by using an in house RIA and Garagorri et al. by using a currently withdrawn RIA assay without extraction, we found that boys had higher A4 concentration than age-matched girls within the first 2 months of life. Despite agreement of NE-A4 reference intervals from our study with concentrations observed by Garagorri et al. [19] for neonates, our present study showed that solvent extraction prior to serum A4 measurement significantly lowered A4 concentrations at this period of life and eliminated sex-related differences thus suggesting the presence of interfering steroids in the neonatal period. Interference was well documented for 17OHP measurement by RIA [3–5]; to our knowledge, no other study has previously investigated the existence of interfering substances for A4 measurement by using RIA during the neonatal period. Of note, the extraction procedure allowed us to increase the specificity of the RIA used for serum A4 quantification in neonates although we were not able to improve the assay sensitivity. Moreover, in a large cohort of children, Kushnir et al. reported A4 reference interval concentrations at least twofold lower than ours for the same period of life, thus reflecting the known higher specificity and sensitivity of gold standard LC-MS/MS assays [13]. In spite of the small number of observations in our cohort, the lack of significant differences between NE-A4 and E-A4 samples in children older than 2 months would suggest that the extraction procedure is not necessary thereafter. It is noteworthy that in children ≤ 2 months, we found that A4 levels were always above the limit of quantification of the RIA-DIAsource method thus suggesting A4 measurement usefulness as an additional circulating steroid marker in newborns in whom a poor steroidogenic activity disorder is suspected. We observed that after the 4th month of age, A4 concentration continued decreasing with CA to reach a nadir during prepuberty until the age of 5. Similar results were obtained by using other methodologies [2, 13, 18, 20]. In prepubertal children, A4 significantly increased in both sexes at the age of 5–8 years. This increment could be explained by the adrenarche, biochemical event with the characteristic increase of SDHEA (exclusive of adrenal synthesis) in which the inner layer of the cortex of the adrenal gland (zona reticularis) is fully developed and secrets androgen of adrenal origin [10]. During peripubertal ages, girls had significantly higher A4 concentration than age-matched boys probably due to the influence of the active GH/IGF-I axis and insulin resistance associated to the mechanism of adrenarche in normal girls and not in boys [21, 22]. Androstenedione concentration was higher in all pubertal groups as compared to that in prepubertal children, becoming evident the contribution of A4 secretion by the gonads. In the literature, there are controversies regarding the impact of sex on A4 concentration in pubertal children [13, 18, 20]. Coinciding with a previous study [18], we were not able to find significant sex-related differences in our pubertal groups. Since A4 concentration in pubertal girls and women varies along the menstrual cycle, our discrepancy with other authors that reported higher A4 levels in females may reside also in the time of sample collection that in our cohort was done always in the early follicular phase [13, 20].

Reference intervals are important for the interpretation of laboratory data. For pediatric settings, it is also essential to know the physiology of intrinsic mechanism underlying each variation to improve diagnostic efficiency at each period of life. In this sense, our study not only provides RI for serum A4 concentration covering the entire pediatric period but also reviews the physiology that accompanies changes in circulating A4 concentration. The influence of the methodological extraction procedure during the neonatal period, as well as age and sexual development throughout prepubertal and pubertal stages, highlights the need to consider all these factors when evaluating A4 results in children in whom steroidogenic disorders are suspected. Because of the lack of confident reference data in the literature of A4 concentration for childhood and adolescence for current commercial RIA methods, our data could be a useful tool in the assessment of A4 concentration in children.

## Figures and Tables

**Table 1:** Androstenedione reference intervals for children from birth up to adolescence.

Group	Androstenedione (nmol/L)
*Full-termchildren*<*1 year of age*	
Nonextracted sera	
Girls ≤ 2 months	3.8 (1.1–7.5)
Boys ≤ 2 months	5.6 (0.5–10.1)
2–4 months	1.4 (1.2–2.6)
4–12 months	0.4 (0.3–1.0)
Extracted sera	
≤2 months	3.1 (0.5–7.6)

*Girls*≥* 1 year*	
Tanner I	
1–5 years	0.3 (0.3–1.0)
5–8 years	1.1 (0.3–2.7)
>8 years	2.7 (1.2–4.7)
Tanner II-III	4.0 (1.8–10.1)
Tanner IV-V	6.2 (3.1–10.9)

*Boys* ≥ *1 year*	
Tanner I	
1–5 years	0.3 (0.3–0.5)
5–9 years	1.3 (0.3–2.8)
>9 years	1.2 (0.3–4.9)
Tanner II-III	3.5 (2.3–6.7)
Tanner IV-V	5.6 (3.0–9.5)

Data are expressed as the median and 3rd–97th centile range.

## References

[1] Speiser P. W., White P. C. (2003). Congenital adrenal hyperplasia. *The New England Journal of Medicine*.

[2] Kyriakopoulou L., Yazdanpanah M., Colantonio D. A., Chan M. K., Daly C. H., Adeli K. (2013). A sensitive and rapid mass spectrometric method for the simultaneous measurement of eight steroid hormones and CALIPER pediatric reference intervals. *Clinical Biochemistry*.

[3] Makela S. K., Ellis G. (1988). Nonspecificity of a direct 17 alpha-hydroxyprogesterone radioimmunoassay kit when used with samples from neonates. *Clinical Chemistry*.

[4] Wong T., Shackleton C. H., Covey T. R., Ellis G. (1992). Identification of the steroids in neonatal plasma that interfere with 17 alpha-hydroxyprogesterone radioimmunoassays. *Clinical Chemistry*.

[5] Ballerini M. G., Chiesa A., Scaglia P., Gruñeiro-Papendieck L., Heinrich J. J., Ropelato M. G. (2010). 17*α*-hydroxyprogesterone and cortisol serum levels in neonates and young children: influence of age, gestational age, gender and methodological procedures. *Journal of Pediatric Endocrinology & Metabolism*.

[6] Ballerini M. G., Chiesa A., Morelli C., Frusti M., Ropelato M. G. (2014). Serum concentration of 17*α*-hydroxyprogesterone in children from birth to adolescence. *Hormone Research in Pædiatrics*.

[7] Domené H. M., Scaglia P. A., Martínez A. S. (2013). Heterozygous IGFALS gene variants in idiopathic short stature and normal children: impact on height and the IGF system. *Hormone Research in Pædiatrics*.

[8] Marshall W., Tanner J. (1969). Variation in pattern of pubertal changes in girls. *Archives of Disease in Childhood*.

[9] Marshall W., Tanner J. (1970). Variation in pattern of pubertal changes in boys. *Archives of Disease in Childhood*.

[10] Belgorosky A., Baquedano M. S., Guercio G., Rivarola M. A. (2008). Adrenarche: postnatal adrenal zonation and hormonal and metabolic regulation. *Hormone Research*.

[11] Nguyen T. V., Nelson A. E., Howe C. J. (2008). Within-subject variability and analytic imprecision of insulinlike growth factor axis and collagen markers: implications for clinical diagnosis and doping tests. *Clinical Chemistry*.

[12] Bruce S. J., Rey F., Béguin A., Berthod C., Werner D., Henry H. (2014). Discrepancy between radioimmunoassay and high performance liquid chromatography tandem-mass spectrometry for the analysis of androstenedione. *Analytical Biochemistry*.

[13] Kushnir M. M., Blamires T., Rockwood A. L. (2010). Liquid chromatography-tandem mass spectrometry assay for androstenedione, dehydroepiandrosterone, and testosterone with pediatric and adult reference intervals. *Clinical Chemistry*.

[14] Bergadá I., Milani C., Bedecarrás P. (2006). Time course of the serum gonadotropin surge, inhibins, and anti-Müllerian hormone in normal newborn males during the first month of life. *The Journal of Clinical Endocrinology and Metabolism*.

[15] Forest M. G., Sizonenko P. C., Cathiard A. M., Bertrand J. (1974). Hypophyso-gonadal function in humans during the first year of life. 1. Evidence for testicular activity in early infancy. *The Journal of Clinical Investigation*.

[16] Forest M. G., Cathiard A. M. (1975). Pattern of plasma testosterone and delta4-androstenedione in normal newborns: evidence for testicular activity at birth. *The Journal of Clinical Endocrinology and Metabolism*.

[17] Mesiano S., Jaffe R. B., Saez J. M., Brownie A. C., Capponi A. (1992). Regulation of growth and function in the human fetal adrenal. *Cellular and Molecular Biology of the Adrenal Cortex*.

[18] Kulle A. E., Riepe F. G., Melchior D., Hiort O., Holterhus P. M. (2010). A novel ultrapressure liquid chromatography tandem mass spectrometry method for the simultaneous determination of androstenedione, testosterone, and dihydrotestosterone in pediatric blood samples: age- and sex-specific reference data. *The Journal of Clinical Endocrinology and Metabolism*.

[19] Garagorri J. M., Rodríguez G., Lario-Elboj A. J., Olivares J. L., Lario-Muñoz A., Orden I. (2008). Reference levels for 17-hydroxyprogesterone, 11-desoxycortisol, cortisol, testosterone, dehydroepiandrosterone sulfate and androstenedione in infants from birth to six months of age. *European Journal of Pediatrics*.

[20] Lo M. S., Ng M. L., Wu L. L., Khalid B. A. (1996). Measurement of androstenedione levels by an in-house radioimmunoassay. *The Malaysian Journal of Pathology*.

[21] Guercio G., Rivarola M. A., Chaler E., Maceiras M., Belgorosky A. (2002). Relationship between the GH/IGF-I axis, insulin sensitivity, and adrenal androgens in normal prepubertal and pubertal boys. *The Journal of Clinical Endocrinology and Metabolism*.

[22] Guercio G., Rivarola M. A., Chaler E., Maceiras M., Belgorosky A. (2003). Relationship between the growth hormone/insulin-like growth factor-I axis, insulin sensitivity, and adrenal androgens in normal prepubertal and pubertal girls. *The Journal of Clinical Endocrinology and Metabolism*.

